# Human stromal cells are required for an anti-breast cancer effect of zoledronic acid

**DOI:** 10.18632/oncotarget.4421

**Published:** 2015-06-10

**Authors:** Hilde H. Nienhuis, Marlous Arjaans, Hetty Timmer-Bosscha, Elisabeth G.E. de Vries, Carolina P. Schröder

**Affiliations:** ^1^ Department of Medical Oncology, University of Groningen and University Medical, Center Groningen, Groningen, The Netherlands

**Keywords:** breast cancer, microenvironment, zoledronic acid, TGF-β, CAM model

## Abstract

Previous studies suggested that bisphosphonate zoledronic acid exerts an anti-tumor effect by interacting with the microenvironment. In this study, we aimed to elucidate the mechanism behind the anti-breast cancer effect of zoledronic acid.

Here we showed that zoledronic acid did not influence *in vitro* human breast cancer cell survival, but did affect human stromal cell survival. Breast cancer cell death in co-culture with stromal cells was analyzed *in vitro* by fluorescent microscopy and flowcytometry analysis. In co-culture, the addition of stromal cells to breast cancer cells induced tumor cell death by zoledronic acid, which was abolished by transforming growth factor (TGF)-β. In the *in vivo* chicken chorioallantoic membrane model, zoledronic acid reduced the breast cancer cells fraction per tumor only in the presence of human stromal cells. Zoledronic acid decreased TGF-β excretion by stromal cells and co-cultures. Moreover, supernatant of zoledronic acid treated stromal cells reduced phospho-Smad2 protein levels in breast cancer cells. Thus, zoledronic acid exerts an anti-breast cancer effect via stromal cells, accompanied by decreased stromal TGF-β excretion and reduced TGF-β signaling in cancer cells.

## INTRODUCTION

Breast cancer accounts for the highest cancer incidence and cancer mortality among women worldwide [1]. Despite great advances in breast cancer treatment, including increasingly targeted systemic treatment, development of metastatic disease still cannot be prevented in all patients. Therefore, there is a continuous search for new treatment strategies that may increase the effect of systemic therapy. Currently, the importance of the tumor microenvironment in which immune cells, fibroblasts, adipose cells and endothelial cells are involved in tumor growth and metastasis has become more evident [2]. Furthermore, preclinical studies have also shown that the microenvironment is an important regulator of cancer cell related drug sensitivity [3]. This provides the rational for targeting not only the cancer cells, but also the tumor microenvironment to improve treatment options for breast cancer patients.

Bisphosphonates, currently used as supportive treatment in breast cancer patients with bone metastases, have a potential anti-cancer effect via microenvironmenal cells. A recent meta-analysis [4] concluded that this class of compounds induces survival benefit in postmenopausal women being treated for breast cancer. Although the anti-cancer effect of bisphosphonates is apparently not limited to their anti-resorptive role in bone lesions [5], the mechanism behind this anti-cancer response remains unclear. Recent research has elucidated parts of this mystery by showing that the bisphosphonates risedronate and pamidronate are internalized by tumor-associated macrophages, but not by mouse tumor cells 4T1 [6]. The authors concluded that these compounds target macrophages, but not tumor cells. These results support an indirect anti-tumor effect of bisphosphonates.

It would be of great benefit for further optimization when this indirect anti-tumor effect would be further clarified. For this, optimal preclinical models containing both human cancer cells and human stromal components are essential [7, 8]. At present optimal preclinical models are lacking. *In vitro* culture models are mostly too simplified and traditional mouse models fall short in this setting, since mouse stromal infiltration into human cell line xenografts as well as into patient derived xenografts occur to a high extent [9, 10].

We have optimized the chorioallantoic membrane (CAM) model, which makes it possible to study the direct interactions between human tumor cells and human stromal cells *in vivo* in an immune deprived setting. By using *in vitro* and *in vivo* models consisting of human stromal cells as well as human breast cancer cells, we studied the role of stromal cells in breast cancer bisphosphonate sensitivity. Our research provides functional evidence of the role of stromal cells in zoledronic acid (ZOL) mediated breast cancer cell death.

## RESULTS

### Stromal cells are required for the anti-breast cancer effect of ZOL *in vitro*

**Figure 1:**
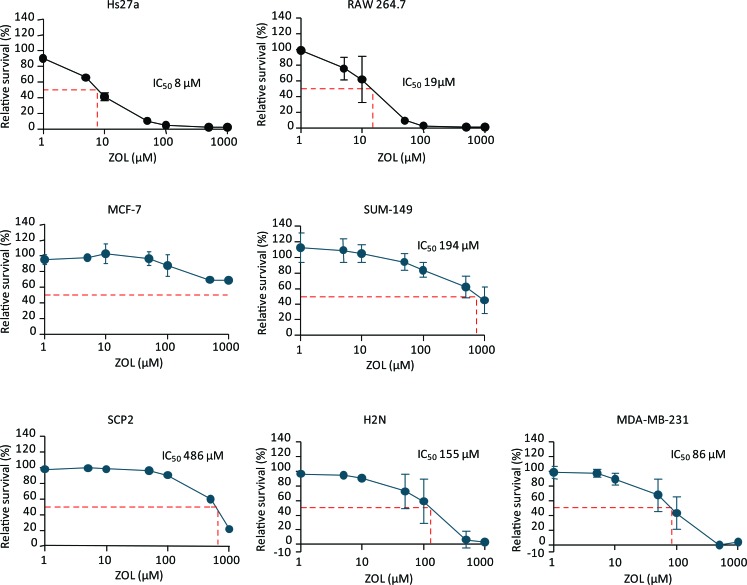
Relative survival after exposure to zoledronic acid Relative survival (%) curves after 96 hours of exposure to 1 – 1000 μM zoledronic acid of the stromal cell lines Hs27a and RAW 264.7 and the breast cancer cell lines MCF-7, SUM-149, SCP2, H2N and MDA-MB-231. The half maximal inhibitory concentration (IC_50_) is depicted for every cell line (dashed red line). Data are represented as mean ± SD.

High concentrations of ZOL were required to decrease cell survival of the human breast cancer cell lines. The MCF-7 line was not sensitive to ZOL treatment. The human breast tumor cell lines SCP2, SUM-149, H2N and MDA-MB-231 had an IC_50_ of 486, 194, 155 and 86 μM respectively. However, ZOL affected stromal cell survival at concentrations far below the concentrations directly affecting breast cancer cell lines: IC_50_ for human stromal cell line Hs27a was reached at a concentration of only 8 μM. In addition, the macrophage cell line RAW 264.7 was also very sensitive to ZOL treatment, with an IC_50_ of 19 μM (Figure 1).

**Figure 2:**
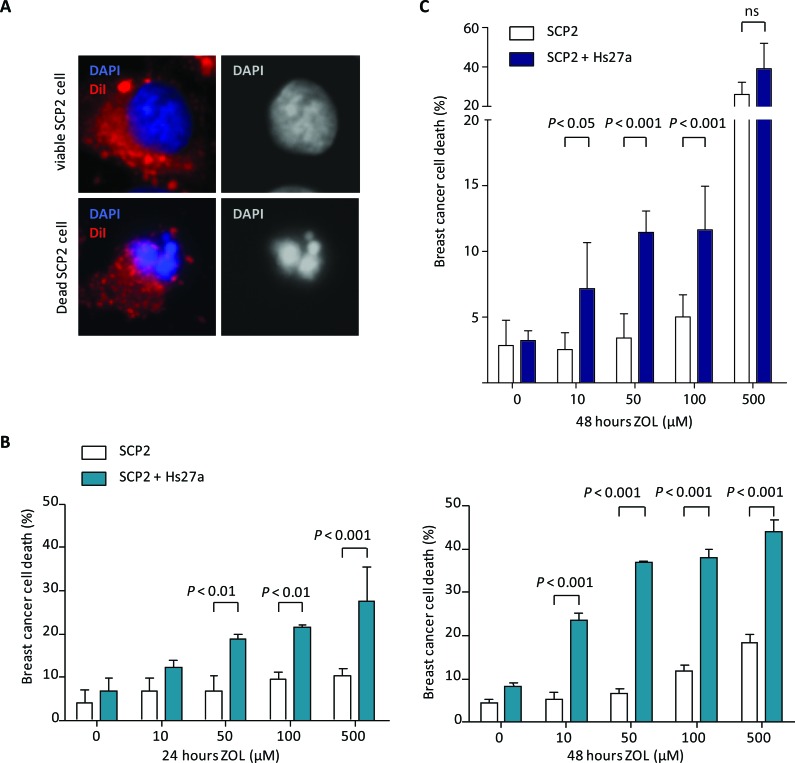
*In vitro* breast cancer cell viability in co-culture after zoledronic acid treatment **A.** Representative images presenting the assessment of SCP2 cell viability by fluorescence microscopy in the *vitro* co-culture model at x 40 magnification. The overlay shows DAPI nuclear staining (blue) and membrane staining with DiI (red). Nuclei of viable SCP2 cells are round and intact, whereas nuclei of dead SCP2 cells are condensed and fragmented. **B.** Viability (%) of SCP2 mono-culture or co-culture with Hs27a stromal cells after 24 and 48 hours of treatment with 0 – 500 μM zoledronic acid analyzed by fluorescent microscopy. **C.** Viability (%) of SCP2 mono-cultures or co-cultured with Hs27a stromal cells after 24 hours of treatment with 0 – 500 μM zoledronic acid determined by flowcytometric measurements of DiI and LIVE/DEAD stain. Data are represented as mean ± SD.

To investigate the indirect effects of ZOL, we used a fluorescence-based *in vitro* co-culture model. In this model, SCP2 cells were fluorescently labeled before addition to an Hs27a monolayer, in order to distinguish tumor cells from stromal cells in cell death assessment. Representative nuclear structures of a viable and a dead SCP2 cell are depicted in Figure 2A. At 24 hours (Figure 2B), 50 μM of ZOL increased breast cancer cell death in the co-culture group (SCP2 and Hs27a) compared to the mono-culture (SCP2) cancer cell group (18.9 ± 1 % *vs* 6.8 ± 3.5 %, *P* < 0.01). This effect was ZOL dose-dependent in the co-culture group, increasing breast cancer cell death to 21.6 ± 0.6 % for 100 μM (*P* < 0.01) and 27.6 ± 7.8 % (*P* < 0.001) for 500 μM. In mono-culture, increasing the dose of ZOL did not increase breast cancer cell death (9.6 ± 1.6 % for 100 μM and 10.3 ± 1.7 % for 500 μM of ZOL). At 48 hours, the stromal-dependent breast cancer cell death induced by ZOL was even more pronounced than at 24 hours (Figure 2B). At a ZOL dose of only 10 μM, breast cancer cell death in the co-culture group (23.5 ± 2.8 %) was higher compared to the mono-culture group (5.1 ± 3.1 %, *P* < 0.001). And the effect became more pronounced as the dose of ZOL increased, with breast cancer cell death of 6.5 ± 2 % for 50 μM, 11.8 ± 2.3 % for 100 μM and 18.4 ± 3.3 % for 500 μM in the mono-culture group versus 37.0 ± 0.4 % for 50 μM, 38.0 ± 3.4 % for 100 μM and 44.0 ± 4.6 % for 500 μM in the co-culture group (*P* < 0.001 for all doses). In mono-cultures of SCP2, ZOL increased breast cancer cell death after 48 hours compared to control from 4.3 ± 1.4 % to 11.8 ± 2.3 % (*P* < 0.05) for 100 μM and 18.4 ± 3.3 % (*P* < 0.001) for 500 μM ZOL (Figure 2B).

Breast cancer cells death after ZOL treatment was also determined by flowcytometry analysis. SCP2 cells were labeled with DiI and cell death was determined by LIVE/DEAD stain uptake. In the presence of stromal cells, SCP2 cell death was induced after treatment with ZOL. At 24 hours (Figure 2C), 10 μM of ZOL increased breast cancer cell death in the co-culture group (SCP2 and Hs27a) compared to the mono-culture (SCP2) group (7.2 ± 3.0% *vs* 2.5 ± 1.1 %, *P* < 0.05). This effect was ZOL dose-dependent in the co-culture group, increasing breast cancer cell death to 11.4 ± 1.4 % for 50 μM (*P* < 0.001), 11.6 ± 2.9 % for 100 μM (*P* < 0.001). For 500 μM no difference was seen in SCP2 cell death with and without Hs27a cells (39.1 ± 10.5 % *vs* 26.1 ± 5.1).

### Stromal cells are required for anti-breast cancer cell effect by ZOL *in vivo*

In the *in vivo* CAM assay, we investigated the effect of ZOL in two different breast cancer suptypes; ER positive (MCF-7) and triple negative (SCP2) breast cancer. Tumors grown on the CAM assay consisted of tumor cells only, tumor cells mixed with Hs27a stromal cells or Hs27a cells only.

**Figure 3:**
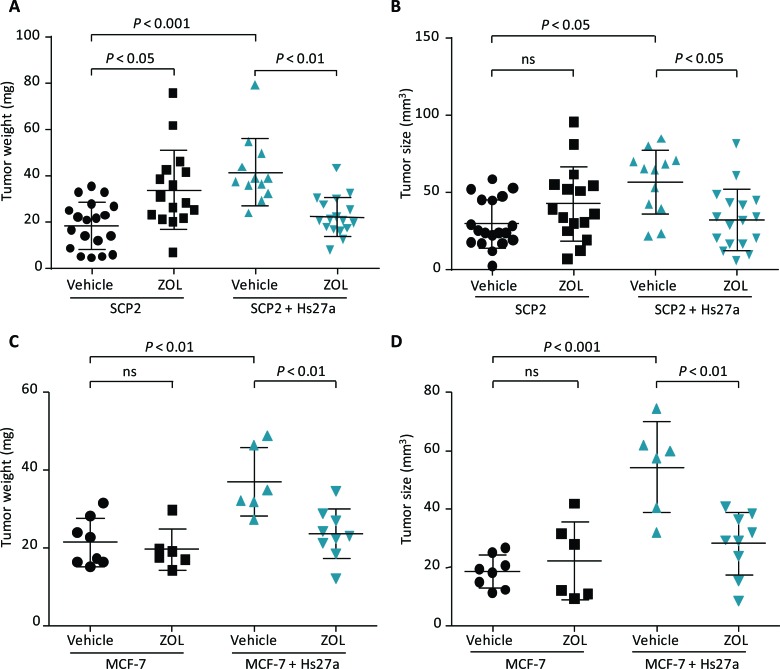
Tumor size and weight after zoledronic acid treatment *in vivo.* Scatter plots illustrating weight (mg) and size (mm^3^) of *in ovo* tumors harvested on day 14 after a single gift of zoledronic acid (200 μM) or vehicle (PBS) on day 10. Tumors consisted of SCP2 alone or SCP2 with Hs27a stromal cells **A.**, **B.** and MCF-7 alone or MCF-7 with Hs27a stromal cells **C.**, **D.**. Tumor weight and size are depicted for every individual tumor. Data are represented as mean ± SD.

On day 14 of the *in vivo* CAM assay, vehicle-treated tumors containing SCP2 plus Hs27a cells were heavier (42.7 ± 14.7 mg *vs* 21.6 ± 10.3 mg, *P* < 0.001) and larger (55.5 ± 21.7 mm^3^ *vs* 31.8 ± 15.5 mm^3^, *P* < 0.05) compared to tumors containing only SCP2 cells (Figure 3A and 3B). Tumors containing only SCP2 cells had a higher weight after treatment with ZOL compared to vehicle (33.9 ± 17.1 *vs* 21.6 ± 10.3 mg (*P* < 0.05). The tumor size was not affected by ZOL compared to vehicle treatment for tumors consisting of only SCP2 cells. However, tumors containing SCP2 plus Hs27a cells were sensitive to ZOL. On day 14, these tumors weighed less and were smaller when treated with ZOL compared to vehicle-treated SCP2 plus Hs27a tumors (tumor weight: 23.0 ± 8.6 mg *vs* 42.7 ± 17.7 mg *P* < 0.01, size: 32.4 ± 19.8 mm^3^ *vs* 55.5 ± 21.7 mm^3^ *P* < 0.05) (Figure 3A and 3B).

ZOL treatment showed similar results for tumors consisting of only MCF-7 cells and tumors containing MCF-7 plus Hs27a cells. On day 14, tumors comprising MCF-7 plus Hs27a cells were heavier (36.9 ± 8.8 mg *vs* 21.4 ± 6.2 mg *P* < 0.01) and larger (54.4 ± 15.5 mm^3^ *vs* 18.6 ± 5.6 mm^3^ *P* < 0.001) (Figure 3C and 3D) compared to tumors consisting of only MCF-7 cells. Moreover, ZOL had no effect on tumors containing only MCF-7 cells. However, in tumors containing MCF-7 plus Hs27a cells, ZOL treatment resulted in reduced tumor weight and size compared to vehicle-treated tumors (weight: 23.5 ± 6.4 mg *vs* 36.9 ± 8.8 mg *P* < 0.01, size: 28.2 ± 10.7 *vs* 54.4 ± 15.5 mm^3^ *P* < 0.01) (Figure 3C and 3D). ZOL did not affect size and weight of tumors containing only Hs27a cells (Suppl. Figure 1).

**Figure 4:**
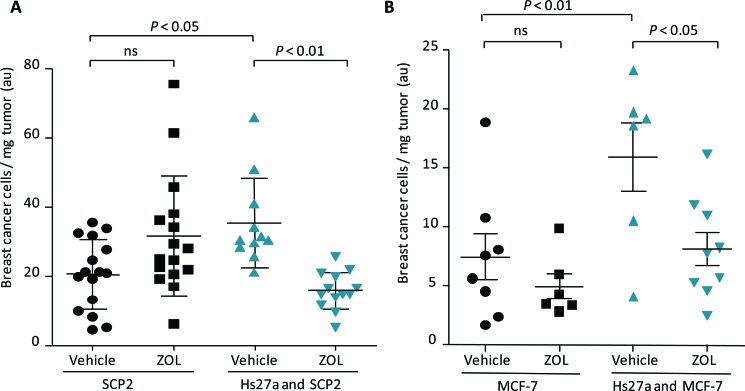
Breast cancer cell fraction after zoledronic acid treatment *in vivo.* Scatter plots illustrating quantified breast cancer cell fraction (breast cancer cells/mg tumor) of *in ovo* tumors harvested on day 14 after a single gift of zoledronic acid (200 μM) or vehicle (PBS) on day 10. Tumors consisted of SCP2 alone or SCP2 with Hs27a stromal cells **A.** and MCF-7 alone or MCF-7 with Hs27a stromal cells **B.**. Breast cancer fraction is depicted for every individual tumor. Data are represented as mean ± SD.

To verify that the reduction in both size and weight of tumors consisting of both breast cancer and stromal cells by ZOL was caused by loss of breast cancer cells, the breast cancer fraction of all tumors was determined. The breast cancer fraction accounts for the ratio of breast cancer cells on the total number of cells, multiplied by the weight of the tumor. The breast cancer cell fraction of tumors containing only SCP2 or MCF-7 cells was not affected by ZOL. However, the breast cancer cell fraction of SCP2 and MCF-7 tumors that also contained stromal cells was larger than SCP2 or MCF-7 cells only. ZOL reduced the breast cancer cell fraction of the co-cultured tumors (*P* < 0.01 SCP2, *P* < 0.05 MCF-7). Moreover, the size of ZOL-treated SCP2 and MCF-7 co-culture tumors was reduced compared to the size of their respective mono-culture tumors (Figure 4A and 4B). Hematoxylin & eosin (H&E) staining showed no difference in tumor viability between the tumor groups (Suppl. Figure 2).

### Reduced TGF-β signaling in stromal cells is required for anti-breast cancer effect of ZOL

**Figure 5:**
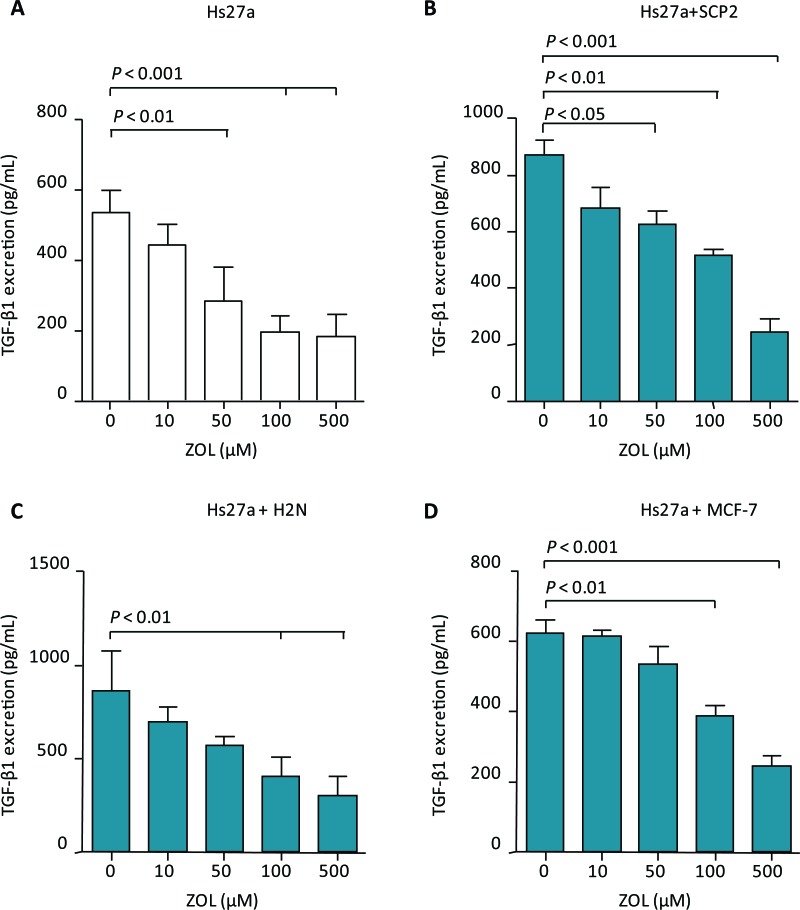
TGF-β1 excretion after 48 hours of zoledronic acid treatment *in vitro.* Total TGF-β1 excretion (pg/mL) after 48 hours of exposure to 0 – 500 μM zoledronic acid of Hs27a stromal cells in mono-culture **A.** or co-cultured with SCP2 **B.**, H2N **C.** or MCF-7 **D.**. Data are represented as mean ± SD.

Total transforming growth factor (TGF)-β1 excretion levels, both active and latent, in supernatant of stromal cells and co-cultures were reduced by 48 hours treatment with ZOL (Suppl. Table 1). Total TGF-β1 levels in supernatant of stromal cells decreased after treatment with 50, 100 and 500 μM ZOL (Figure 5A). In the supernatant of SCP2 co-cultured with Hs27a, total TGF-β1 levels also decreased after treatment with ZOL 50 μM, 100 μM and 500 μM (Figure 5B). In co-cultures of H2N with Hs27a and MCF-7 with Hs27a, total TGF-β1 levels were reduced after treatment of 100 μM and 500 μM ZOL (Figure 5C and 5D). However, 48 hours of ZOL treatment did not reduce total TGF-β1 levels in the supernatant of SCP2 and H2N breast cancer cells in the absence of stromal cells. In the supernatant of MCF-7 breast cancer cells without stromal cells, total TGF-β1 levels were undetectable (< 0 ± 60 pg/mL) (data not shown). One day of ZOL treatment did not alter total TGF-β1 levels in the supernatant of monocultures of stromal Hs27a cells and SCP2, H2N and MCF-7 cells. This was also the case for the total TGF-β1 levels in the supernatant of co-cultures of stromal and breast cancer cells treated for 24 hours with ZOL (Suppl. Figure 3).

**Table 1:** TGF-β1 excretion after 48 hours of zoledronic acid treatment *in vitro*

Cell lines		Treatment	TGFβ levels		
Cancer	Stroma	ZOL (μM)	Value (pg/mL)	Relative level (%)	*P*
-	Hs27a	0	**538.4** ± 62.8	**100**	ref
		10	**446.4** ± 57.3	**83** ± 10.6	ns
		50	**284.5** ± 96.6	**53** ± 18.1	< 0.01
		100	**197.7** ± 46.8	**37** ± 8.7	< 0.001
		500	**185.9** ± 60.3	**35** ± 11.2	< 0.001
SCP2	Hs27a	0	**871.7** ± 92.3	100	ref
		10	**683.3** ± 124.4	**78** ± 7,2	ns
		50	**628.3** ± 73.9	**72** ± 6.1	< 0.05
		100	**514.3** ± 36.7	**59** ± 7.5	< 0.01
		500	**244.3** ± 80.2	**28** ± 10.2	< 0.001
H2N	Hs27a	0	**866.3** ± 208.0	**100**	ref
		10	**699** ± 74.8	**80** ± 10.4	ns
		50	**575.3** ± 45.3	**66** ± 20.0	ns
		100	**407** ± 98.8	**47** ± 21	< 0.01
		500	**301.7** ± 101.3	**35** ± 3.2	< 0.01
MCF-7	Hs27a	0	**624.7** ± 59.5	**100**	ref
		10	**612.7** ± 33.4	**70** ± 12.0	ns
		50	**535.5** ± 83.05	**61** ± 20.8	ns
		100	**389.0** ± 48.1	**45** ± 11.4	< 0.01
		500	**243.7** ± 55.4	**28** ± 5.6	< 0.001

**Abbreviations:** Zoledronic acid (ZOL), transforming growth factor (TGF)β

**Figure 6:**
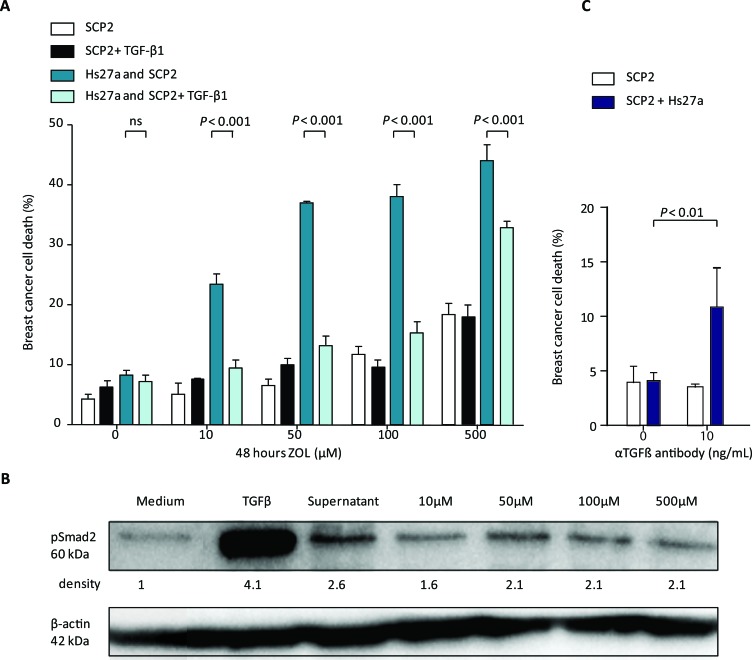
Breast cancer cell death and TGF-β signaling after stromal treatment of zoledronic acid *in vitro.* **A.** Viability (%) of SCP2 mono-culture or co-culture with Hs27a stromal cells after 48 hours of treatment with 0 – 500 μM zoledronic acid with or without pure active TGF-β1 (1 ng/mL) quantified by fluorescent microscopy. **B.** Western blotting of pSmad2 was performed on lysates of SCP2 cells. β-actin serves as a loading control. SCP2 cells were incubated for 1 hour with medium, pure active TGF-β1 (5 ng/mL), or supernatant of Hs27a cells treated for 48 hours with 0 – 500 μM zoledronic acid. The relative density of the pSmad2 bands compared to the loading control is shown. **C.** Breast cancer cells death (%) of SCP2 mono-cultures or co-cultured with Hs27a stromal cells after 24 hours of treatment with anti- TGF-β antibody (10 ng/mL), determined by flowcytometric measurements of DiI and LIVE/DEAD stain. Data are represented as mean ± SD.

ZOL treatment resulted in dose-dependent increased death of SCP2 cells in co-culture with Hs27a cells *in vitro* (Figure 6A). The addition of pure active TGF-β1 to the *in vitro* co-culture model of SCP2 and Hs27a cells treated with ZOL almost completely abolished the ZOL-induced SCP2 cell death. Two days after the addition of pure active TGF-β1, SCP2 cell death decreased from 24.3 ± 2.9 % to 9.5 ± 2.3 % (*P* < 0.001) for 10 μM ZOL, 35.6 ± 2.9 % to 13.3 ± 2.5 % (*P* < 0.001) for 50 μM ZOL, 37.7 ± 2.9 % to 15.3 ± 3.8 % (*P* < 0.001) for 100 μM ZOL and 47.8 ± 8.6 % to 32.9 ± 1.9% (*P* < 0.001) for 500 μM ZOL. The addition of pure active TGF-β1 to monocultures of SCP2 cells treated with ZOL did not have this effect.

Blocking of all TGF-β present in our culture models by a pan-TGF-β antibody resulted in SCP2 cell death only in the presence of Hs27a cells. At 24 hours of TGF-β antibody treatment, breast cancer cell death in co-culture with Hs27a was induced compared to untreated cells (10.9 ± 3.6% *vs* 4.06 ± 0.8% *P* < 0.01) (Figure 6C). In SCP2 mono-cultures, TGF-β antibody treatment did induce SCP2 cell death compared to untreated SCP2 mono-cultures.

Active TGF-β signaling activity in SCP2 breast cancer cells, measured by pSmad2 levels, was increased after incubation with Hs27a supernatant (Figure 6B). As a control, an excess of pure active TGF-β1 (5 ng/mL) was added to the SCP2 cells and this also resulted in clear Smad2 phosphorylation. In contrast, supernatant of Hs27a cells treated with all dosages of ZOL decreased pSmad2 levels. This indicates that ZOL can indirectly reduce active TGF-β signaling in breast cancer cells, via an effect on stromal cells.

## DISCUSSION

Our study provides functional proof that the bisphosphonate ZOL can exert an anti-breast cancer effect only in the presence of stromal cells. We investigated this in various breast cancer subtypes and two microenvironmental cell lines. These results complement research by Junankar et al. showing that the bisphosphonates risedronate and pamidronate target macrophages in the breast cancer microenvironment of mouse 4T1 xenografts [6]. In their mouse model, intravital imaging revealed bisphosphonate uptake in calcium-rich tumor regions, while no uptake was seen in non-cancerous mouse mammary glands. Together with the data by Junankar et al., our study supports the concept of a microenvironment-mediated anti-tumor effect of bisphosphonates, and further clarifies the mechanism behind this effect.

These results suggest that bisphosphonate treatment not only modulates the bone environment, but also affects non-bone disease. This is in line with clinical data. A recent meta-analysis showed that breast cancer recurrence decreased in early breast cancer patients treated with a bisphosphonate [4]. The clear interaction of ZOL with stromal cells in our study supports the broader effect of bisphosphonates outside of the bone environment. For future studies it will be of great interest to further study the anti-breast cancer effect of zoledronic acid exerted via other microenvironmental cell types. Furthermore, it will be relevant to evaluate these effects in a tissue specific context resembling for example the microenvironment of the primary tumor or a specific metastatic site.

Bisphosphonates inhibit osteoclast activity by inhibiting protein prenylation [11]. As inhibition of prenylation play would result in similar effects on breast cancer cell death, with and without stromal cells, this mechanism probably is not responsible for breast cancer cell death in our study. In our co-culture experiments, the reduced difference between breast cancer cells with an without stromal addition at the highest zoledronic acid concentration could be attributed to prenylation effects on the tumor cells. This is then in accordance with the IC_50_ levels observed in the MTT assay.

In our co-culture model, treatment with zoledronic acid led to reduction of total TGF-β levels and active signaling activity. Moreover, treatment of breast cancer cells with an anti-TGF-β antibody instead of zoledronic acid induced breast cancer cell death only in the co-culture condition. These results indicate that reduced TGF-β signaling in breast cancer cells, as observed in co-culture after zoledronic acid treatment, can cause increased cell death of breast cancer cells. However, our results do not rule out that, apart from TGF-β, other factors could also be involved in this tumor-stroma interaction after zoledronic acid treatment. Moreover, the reason to why breast cancer cells, in co-culture compared to mono-culture after zoledronic acid treatment, are more dependent on TGF-β signaling remains to be elucidated. Previous research suggested a strong role for the microenvironment with respect to bisphosphonate sensitivity and TGF-β signaling [12]. In mice, bisphosphonate pamidronate treatment applied to established in-bone metastases of the cell line SCP28 evidently reduced active TGF-β signaling in these metastases. In that study, however, treatment of SCP28 tumor cells with pamidronate *in vitro* – without the presence of stromal cells – affected neither TGF-β signaling nor tumor cell death. Our study explains this apparent paradox by demonstrating the mediating role of the stromal cells. TGF-β transcriptional activity can also be inhibited by ER activation [13], thereby linking our research with pre-clinical and clinical studies showing that bisphosphonates only exert an anti-tumor effect under low estradiol concentrations [14, 15]. If, under high estradiol levels, TGF-β signaling is already repressed, adding another TGF-β suppressing agent like ZOL would have no additional anti-cancer effect.

To our knowledge, ours is the first study to evaluate the effect of ZOL on the breast cancer-stroma interaction by assessing the separate components. This analysis was made possible by using the CAM model. Due to its low immunogenicity, xenografts can be grown on the CAM model up to day 18 of embryonic development [16]. Moreover, in the CAM model, growth of host stroma into the human xenograft is limited. In contrast, tumors of mice inoculated with human cell lines or patient-derived xenografts consist of around 40% mouse DNA [10, 17]. We observed good experimental reproducibility of the CAM assay between separate experiments.

In conclusion, the anti-breast cancer effect of ZOL is dependent on the presence of stromal cells. This is accompanied by decreased stromal cell TGF-β excretion and reduced TGF-β signaling in cancer cells.

## MATERIALS AND METHODS

### Cell lines and reagents

The human cell lines used in this study were representing different breast cancer subtypes and two microenvironmental cell lines. ER positive breast cancer cell line MCF-7 and immortalized stromal cell line Hs27a (American Type Culture Collection (ATCC)) were cultured in Roswell Park Memorial Institute (RPMI) medium (Invitrogen), supplemented with 10% fetal calf serum (FCS). Hs27a cells were cultured up to a maximum of 30 passages during which these cells remained phenotypically stable and viable. Macrophage cell line RAW 264.7 was cultured in Dulbecco's Modified Eagle Medium (DMEM) (Invitrogen) with 10% FCS and 1% glutamine. The inflammatory triple negative breast cancer cell line SUM-149 (Asterand) was cultured in HAM's Nutrient Mixture-F12 (HAM) supplemented with 5% FCS, 5 μg/mL insulin and 1 μg/mL hydrocortisone. Triple negative breast cancer cell line MDA-MB-231 (ATCC), the daughter cell line MDA-MB-231-H2N (H2N) (transfected to stably overexpress HER2) [18], luciferase transfected MDA-MB-231-SCP2 (SCP2) (provided by Dr Y Kang, Princeton University, NJ) [19] were cultured in DMEM with 10% FCS and 1% glutamine. Cell lines were cultured at 37 °C in a humidified atmosphere containing 5% CO_2_ and were routinely tested for *Mycoplasma*. Short tandem repeat profiling (BaseClear) was used to authenticate the cell lines.

### Breast cancer and stromal cell proliferation *in vitro* in response to ZOL

Hs27a, RAW 264.7, MCF-7, MDA-MB-231, H2N, SCP2 and SUM-149 cells were plated in 96-well plates with a density of 2,000 – 12,500 cells/well. ZOL (SelleckChem) was added in different concentrations (1-1,000 μM) and cells were incubated for 96 hours. Thereafter, 3-(4,5-dimethylthiazol-2-yl)-2,5- diphenyltetrazolium bromide (MTT) was added and formazan production as read out of cell number and metabolic activity was measured as described previously [20].

### Breast cancer cell death in an *in vitro* co-culture model in response to ZOL

This fluorescence-based *in vitro* co-culture model was described previously [21]. To distinguish the cancer cells from the stromal cells, SCP2 cells were pre-labeled with fluorescent marker DiI (Molecular Probes, Invitrogen). Hs27a cells were grown as monolayer on glass slides inserted in 24-well plates. Subsequently, 15,000 SCP2 cells were added per well, with or without a pre-cultured stromal cell monolayer and allowed to attach to the glass insert or the stromal layer for 24 hours. Attached cells were treated with ZOL (10 - 500 μM) for 24 or 48 hours. To study whether the effects of ZOL could be counteracted by TGF-β1, a condition with active TGF-β1 addition was included. The cells were cultured for 48 hours following the addition of 1 ng/mL pure active TGF-β1 (PeproTech) concurrently with ZOL.

After incubation, the plates were centrifuged at 300 g for 7 minutes. Glass slides were collected, fixed with methanol:acetone (1:1) and stained with 1:1000 4′,6-diamidino-2-phenylindole (DAPI). The results were analyzed by fluorescent microscopy. Tumor cell viability was assessed with nuclear DAPI staining based on observation of the nuclear structure (intact *versus* fragmented nuclei). For each condition, 6 - 12 fields of view were counted and the average percentage of dead cells was calculated.

Breast cancer cell death after ZOL treatment in presence and absence of stromal cells was determined by an additional method. Hs27a cells were grown as monolayer in 6-well plates. SCP2 cells were pre-labeled with fluorescent marker DiI and plated, with a cell density of 200,000 cells per well, on plates with or without pre-cultured stromal layer. Attached cells were treated with ZOL (10 - 500 μM) or a pan-TGF-Δ antibody (10 ng/mL) (R&D systems) for 24 hours. Cell death was determined by flowcytometry analysis after incubation with fluorescent LIVE/DEAD stain (Life Technologies) by manufacturer's protocol as described before [22]. Cells positive for DiI and LIVE/DEAD stain were considered as dead breast cancer cells. Samples were acquired with FACS LSR II (Becton-Dickinson) and the percentage of double positive cells (dead breast cancer cells) compared to DiI positive cells (all breast cancer cells) was quantified by FlowJo (Tree Star, Inc.).

### Breast cancer cell death in an *in vivo* co-culture model in response to ZOL

To study the effect of ZOL *in vivo*, the CAM model was used as described previously [23]. Two breast cancer models, SCP2 and MCF-7, were studied in this way. Eggs (het Anker BV) were incubated at 38°C and after 3 days the CAM was lowered by puncturing the top of the eggs. On day 6 of embryonic development, a window was made in the egg shell to access the CAM. The CAM was damaged using a cotton tissue (Celltork) and each egg was inoculated with a total of 5·10^6^ cells in 50 μL culture media and Matrigel (BD Biosciences) (1:1). On day 10 of embryonic development, a 4 mm latex ring (Dentsply International) was placed on the CAM and 15 μL of ZOL (200 μM) or vehicle was pipetted in the ring. The dose ZOL used in the CAM assay was based on our *in vitro* experiments and concurs with a dosage of 16 μg/kg for an egg weighing on average 0.05 kg. This is a low dose compared to the clinic, where a patient receives a dose of 4 mg concurring with a dosage of 53 μg/kg for a patient weighing 75 kg.

Each experiment comprised 3 subgroups; group 1) breast cancer cells only, SCP2 or MCF-7 cells treated with ZOL (SCP2 *n* = 15, MCF-7 *n* = 6) or vehicle (SCP2 *n* = 17, MCF-7 *n* = 8), group 2) Hs27a cells treated with ZOL (*n* = 8) or vehicle (*n* = 7) and group 3) a mixture of breast cancer cells, SCP2 or MCF-7, with Hs27a cells (1:1) treated with ZOL (SCP2 *n* = 17, MCF-7 *n* = 9) or vehicle (SCP2 *n* = 12, MCF-7 *n* = 6). On day 14 of embryonic development, tumors were harvested, weighed and measured with a caliper.

Tumor tissue was formalin-fixed and paraffin embedded for immunohistochemical analyses. H&E staining was performed to analyze tissue viability and morphology. Slides (5 μm) were stained with an antibody against cytokeratin (CK) 8, 18 and 19 (Abcam 1:100). Substitution of the 1^st^ antibody by bovine serum albumin was used as negative control. To calculate the breast cancer cell fraction in the tumors, the ratio of CK positive cells on the total number of cells was determined per tumor. Then this number was multiplied by the total tumor weight.

### Stromal and breast cancer TGF-β1 excretion in mono- and co-culture models *in vitro* in response to ZOL

Total TGF-β1 levels were determined in supernatant of Hs27a cells in mono-culture or in co-culture with MCF-7, SCP2 or H2N cells after 24 and 48 hours incubation with ZOL. Cell culture supernatant was removed, centrifuged for 15 minutes at 240 g to remove any residual cells or cell remnants, and subsequently stored frozen in aliquots. Total TGF-β1 levels were measured with enzyme-linked immunosorbent assays (ELISA) according to manufacturer's instructions (Quantikine, R&D Systems). The absorbance of each well was measured by a microplate reader (Bio-Rad).

### Breast cancer TGF-β signaling *in vitro* in response to ZOL

As read out for TGF-β signaling activity, protein expression of phosphorylated Smad2 was measured by Western blot. Hs27a cells were grown as monolayer and treated with ZOL (10 - 500 μM) for 48 hours. Subsequently, 500,000 SCP2 cells were plated in 6 wells plates and incubated for 24 hours. Pure active TGF-β1 (5 ng/mL) or harvested supernatant of Hs27a cells was added to the confluent SCP2 cells. After 1 hour incubation, the supernatant was removed. Total cell lysates were size fractionated and transferred to a membrane as described previously [24].

Membranes were exposed to primary antibodies (anti-pSmad2; Cell Signaling Technology, anti-β-actin; MP Biomedicals). Binding of antibodies was determined using horseradish peroxidase (HRP)-conjugated secondary antibodies (DAKO) and visualized with Lumi-light^plus^ (Roche Diagnostics). Band density was evaluated by ImageJ software.

### Statistical analysis

Data are presented as mean ± SD. Statistical analysis was performed using the one-way or two-way ANOVA test with Tukey's or Bonferroni's post hoc test (GraphPad Prism, version 5). Differences were considered significant when *P* < 0.05.

## SUPPLEMENTARY MATERIAL FIGURES


