# Supplementary material for: Human stromal cells are required for an anti-breast cancer effect of zoledronic acid
Source: Oncotarget. 2015 Jun 10;6(27):24436–47. doi: 10.18632/oncotarget.4421 (PMC4695196; doi:10.18632/oncotarget.4421)
Supplement: Supplementary file 1 [file oncotarget-06-24436-s001.pdf]

Human stromal cells are required for an anti-breast cancer effect of zoledronic acid

Supplementary Material

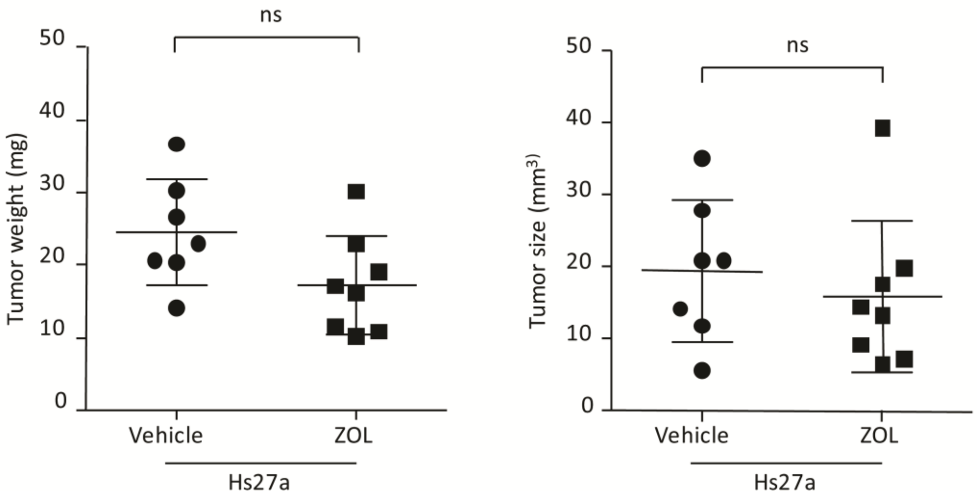

Supplemental figure 1: Stromal cell tumor size and weight after zoledronic acid treatment *in vivo*

Scatter plots illustrating weight (mg) and size (mm^3) of *in ovo* tumors harvested on day 14 after a single gift of zoledronic acid (200 µM) or vehicle (PBS) on day 10. Tumors consisted of Hs27a stromal cells. Tumor weight and size are depicted for every individual tumor. Data are represented as mean ± SD.

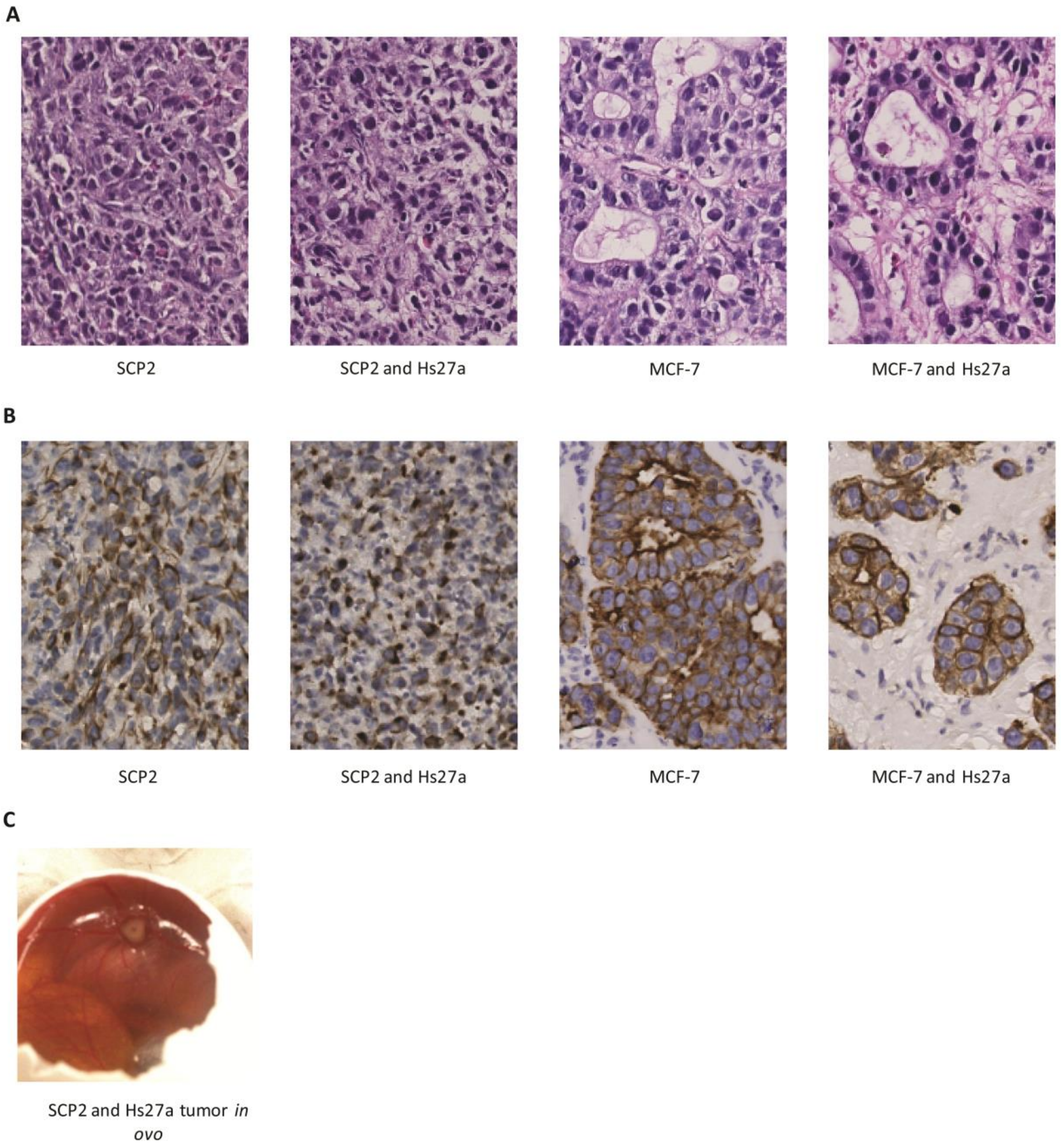

Supplemental figure 2: H&E and cytokeratin staining of tumor tissue

Representative images of H&E **(A)** and cytokeratin staining **(B)** of SCP2, SCP2 and Hs27a, MCF-7, MCF-7 and Hs27a *in ovo* tumors on day 14 at 20x magnification. Images depicted represent vehicle treated tumors. **(C)** *In ovo* SCP2 and Hs27a tumor on day 14 in the CAM model.

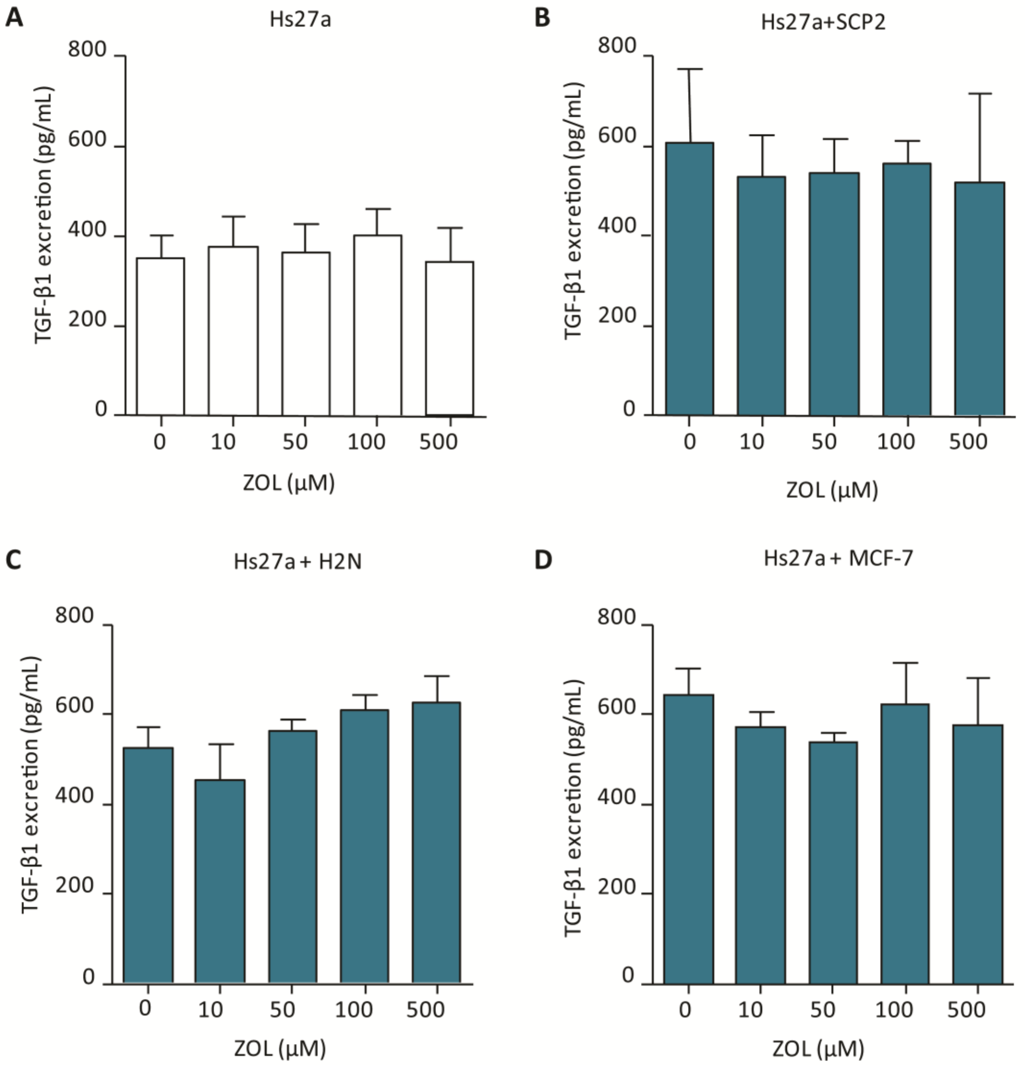

Supplemental figure 3: TGF-β1 excretion after 24 hours of incubation with zoledronic acid

Total TGF-β1 excretion (pg/mL) after 24 hours of exposure to 0 – 500 µM zoledronic acid of Hs27a stromal cells in mono-culture **(A)** or co-cultured with SCP2 **(B)**, H2N **(C)** or MCF-7 **(D)**. Data are represented as mean ± SD.
